# Improving the microbial quality and sensory properties of pasteurized sweet cream butter during refrigerated storage using chia seed ethanolic extract

**DOI:** 10.1002/fsn3.3802

**Published:** 2023-10-31

**Authors:** Zahra Sadri‐Saeen, Mohammadreza Khani, Vajiheh Fadaei

**Affiliations:** ^1^ Department of Food Science and Technology, Shahr‐e‐Qods Branch Islamic Azad University Tehran Iran

**Keywords:** alcoholic extract, butter, chia seeds, microbial quality, sensory properties

## Abstract

Butter is a perishable food, and its microbial deterioration occurs in refrigeration conditions, leading to a reduction in shelf life and a loss of sensory properties. This study aimed to investigate the effect of chia seed extract (CSE) on the microbial and sensory properties of pasteurized sweet cream butter at 2°C. Ethanolic extract of chia seeds was prepared and added to the butter samples in concentrations of 0.05%, 0.1%, 0.25%, and 0.5% (v/w), and its effects on microbiological and sensory quality were evaluated at 15‐day intervals during 60‐day refrigerated storage. The results indicated that the addition of 0.25% and 0.5% CSE to butter treatments decreased total viable counts by 0.25–0.6 log CFU/g, total psychrotrophic counts by 1–1.5 log CFU/g, and coliform counts by 3–4 log CFU/g compared to the control sample on days 45 and 60 of storage. Moreover, concentrations of 0.1%–0.5% CSE reduced mold and yeast counts by 1.5–2.6 log CFU/g on days 30–60 of storage compared to the control sample. *Escherichia coli* and *Staphylococcus aureus* were not detected in any samples during storage. CSE had a significant effect on the sensory properties (except for aroma) of the butter samples during the 60‐day storage. The best color, flavor, and overall acceptance scores were assigned to the treatments containing 0.1%–0.5% CSE compared to the control sample. It could be concluded that adding 0.1% and 0.25% CSE to refrigerated and pasteurized butter can retard microbial spoilage and improve its sensory properties at the same time.

## Graphical abstract

This study highlights how chia seed extract can preserve butter from microbial spoilage and prolong its shelf life in refrigeration conditions. Our results have shown that chia seed extract was not only able to control the microbial growth in butter, but it could also improve its sensory characteristics, including color and flavor, which can increase the palatability of the product.

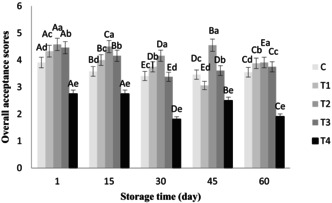

## 1 INTRODUCTION

Butter is a fat‐based dairy product and a water‐in‐oil emulsion in which the water droplets are dispersed and crystallized in the fat phase (Méndez‐Cid et al., 2017). Butter is produced from sweet or sour cream as the main ingredient, which is converted from oil‐in‐water to water‐in‐oil emulsion by a churning process at high speed (Ewe & Loo, 2016). Butter contains approximately 80% fat and 20% water, protein, and carbohydrate, which provides a suitable medium for microbial growth (El‐Aidie, 2018).

Butter made from unsalted sweet cream has the highest vulnerability to microbial contamination in conditions of contaminated water supplies, improper personal hygiene of workers, temperature abuse, and humidity above 70% (Budhkar et al., 2014). Molds are among the initial agents known to cause spoilage in butter, which mainly belong to the genera Thamnidium, Cladosporium, Geotrichum, Penicillium, and Aspergillus (Gazu et al., 2018). Enterobacteriaceae family, coliforms, and other bacteria are responsible for the undesirable color and flavor of butter. Psychrotrophic bacteria, including Pseudomonas spp., such as *P. fragi* and *P. fluorescens*, grow on butter surfaces at 4–7°C and produce extracellular enzymes such as protease, phosphatase, and lipase, which cause putridity and hydrolytic rancidity in butter. Some of these enzymes may remain active in dairy products even after their producing microbes have been destroyed (Ahmed et al., 2016). In addition, some foodborne pathogenic bacteria, including *Listeria monocytogenes*, *E. coli*, and *S. aureus*, have been isolated from butter (Gazu et al., 2018).

For products such as butter that are not exposed to a thermal process, various methods, such as using chemical additives and gamma radiation, have been employed to increase the shelf life of butter, which has caused undesirable changes in some of its chemical and sensory properties (Santos et al., 2017). As a result, the use of natural preservatives has attracted great interest in recent years, and various studies have been conducted to improve the microbial properties of butter (Alipour et al., 2023; Asadaii et al., 2020; Mahmoudi et al., 2019; Thakaeng et al., 2020).

Chia (*Salvia hispanica* L.) is an herbaceous plant that belongs to the *Lamiaceae* family and is native to southern Mexico and northern Guatemala (Khalid et al., 2023). Chia seeds have a high nutritive value because they contain proteins (15%–25%), fats (30%–33%), carbohydrates (26%–41%), dietary fiber (18%–30%), minerals (4%–5%), and vitamins (Kulczyński et al., 2019). Chia seeds also contain a high amount of phenolic compounds with antioxidant activities and polyunsaturated fatty acids such as α‐linolenic acid (omega‐3) and linoleic acid (omega‐6) (Faid, 2017).

Chia seed consumption has beneficial health effects due to its high contents of protein and essential amino acids, essential fatty acids, minerals such as calcium and iron, vitamins, antioxidants, polyphenols, and dietary fiber (Khalid et al., 2023). Because of the health attributes and bioactive potential of chia seed, it has been used as an ingredient in various forms for enriching foods and producing functional foods including bread, yogurt, cheese, ice cream, jam, burger, and sausage (Faid, 2017; Feizi et al., 2021; Kibui et al., 2018; Kwon et al., 2019; Limam & Mohamed, 2019; Nduko et al., 2018; Sandri et al., 2017; Zaki, 2018). Moreover, chia oil and extract have been successfully used to retard oxidative spoilage in butter (Sadri Saeen et al., 2022; Ullah et al., 2020).

The phenolic compounds derived from chia seeds also have antimicrobial activities against Gram‐positive bacteria (such as *S. aureus*, *L. monocytogenes*, and *Clostridium butyricum*), Gram‐negative bacteria (including *E. coli*, *Salmonella typhimurium*, *Salmonella enteritidis*, *Yersinia enterocolitica*, *Shigella dysentery*, *Pseudomonas aeruginosa*, and *Vibrio cholera*), and fungal pathogens, e.g., *Candida albicans* (Kobus‐Cisowska et al., 2019). However, no study has yet evaluated the effects of chia seed on the microbial properties of butter. Therefore, considering that microbial activities cause spoilage in butter, this research was conducted to study the effects of chia seed extract (CSE) on the microbial and sensory properties of pasteurized butter made from sweet cream during refrigerated storage.

## 2 MATERIALS AND METHODS

### 2.1 Materials

The chia seed used in this research was produced in Bolivia, and according to the supplier, it had the following characteristics: Each 100 g had 490 kcal of energy, 34.4 g of fiber, 31.5 g of fat, 16.54 g of protein, 631 mg of calcium, 860 mg of phosphorus, 335 mg of magnesium, 407 mg of potassium, 1.6 mg of vitamin C, 0.5 mg of vitamin E, and 54 IU of vitamin A. All culture media, chemicals, and reagents used in this study were purchased from Merck Company (Darmstadt, Germany).

### 2.2 Preparing alcoholic extract of chia seeds

Ethanolic extract of chia seeds was prepared by grinding them and adding the powder to 70% ethanol (5% w/v) with stirring the solution on a shaker (IKA, MS3 basic, Germany) at 200 rpm for 5–6 h. Then, the solution was kept at room temperature for 24 h and filtered using Whatman filter paper. The filtered extract was concentrated using a rotary evaporator (Heidolph, Laborota 4001, Germany) under vacuum conditions at 60°C for ethanol evaporation (Kwon et al., 2019). The concentrated mixture was dried in an oven (Iran Khodsaz Co., OD 115p, Iran) and then kept in sterile amber glass containers in a refrigerator (Samsung, RT791BATS, Korea) at 4°C until it was used.

### 2.3 Measuring total phenolic content

The total phenolic content (TPC) in CSE was measured based on the method described by Laczkowski et al. (2018). Distilled water (2370 μL) and Folin–Ciocalteu reagent (150 μL) were added to the extract (30 μL) in a test tube and mixed with a vortex for 10 s. A control was prepared by replacing the extract with ethanol (30 μL). After 2 min, sodium carbonate 15% (450 μL) was added, followed by vortex mixing for 10 s. Then, the solutions were kept away from light and incubated for 2 h at room temperature. The absorbance was measured at a wavelength of 765 nm using a spectrophotometer (Unico, UV‐2100, Spain). A calibration curve of gallic acid in the concentration range of 30–1500 mg/L was used to determine TPC. The results were expressed as mg gallic acid equivalents per gram of the sample (mg GAE/g).

### 2.4 Preparing butter samples

Cream with standardized fat content was pasteurized at 85°C for 15 s. It was then stored at 5°C for 72 h to facilitate milk fat crystallization. The cream (4.5–5 kg) was then churned at 7°C using a butter‐making machine (EGLI, EKB2‐IEP, Switzerland) at 150 rpm until the fragile membranes surrounding the milk fat were ruptured and butter grains were formed. The buttermilk was separated from the butter grains, and the butter was washed three times in the churn with potable water by cold (10°C) reverse osmosis. The butter working was done for several minutes at 30 rpm to reduce free water content (O'Callaghan et al., 2016). Then, the produced butter was separately mixed with 0.05%, 0.1%, 0.25%, and 0.5% of CSE (v/w) to obtain homogenized samples. The control sample (C) and the treatments containing CSE (T_1_, T_2_, T_3_, and T_4_, respectively) were packaged in 100 g packages made of polypropylene under vacuum conditions. All samples were kept in the refrigerator at 2°C for 60 days and evaluated in triplicate for microbial and sensory properties on days 1, 15, 30, 45, and 60 of the storage.

### 2.5 Microbiological tests

The initial suspension of butter and decimal dilutions were prepared using Ringer's solution ¼ strength according to ISO 6887‐5 (2010). Total viable counts (TVC) were determined by using the pour plate culture technique on plate count agar (PCA) and incubating plates at 30°C for 72 h according to ISO 4833‐1 (2013). The surface culture method on PCA and aerobic incubation for 10 days at 6.5°C were used according to ISO 17410 (2019) to determine total psychrotrophic counts (TPC). The pour plate method on yeast extract/dextrose/oxytetracycline agar and incubation for 5 days at 25°C according to ISO 6611 (2004) were used to enumerate molds and yeasts. These microbial counts were reported as log10 CFU/g. Coliforms and *E. coli* counts were determined using the most probable number (MPN) method on lauryl sulfate tryptose broth and incubation at 37°C for 24–48 h, followed by confirmatory biochemical tests based on ISO 4831 (2006) and ISO 7251 (2005), respectively. For the detection and enumeration of *S. aureus*, the MPN method on Modified Giolitti and Cantoni broth and aerobic incubation at 37°C for 24 h were employed, followed by confirmatory biochemical tests based on ISO 6888‐3 (2003).

### 2.6 Sensory evaluation

Fifteen untrained panelists were randomly selected to evaluate sensory properties, including flavor, aroma, texture, color, and overall acceptance of all the butter samples on days 1, 15, 30, 45, and 60 of storage using a 5‐point hedonic scale. The panelists expressed their preferential views by giving scores of 1, 2, 3, 4, and 5 for very bad, bad, fair, good, and very good quality, respectively. The samples were removed from the refrigerator 1 h before evaluation to reach a temperature of 13°C (Santos et al., 2017).

### 2.7 Statistical analysis

A completely randomized design was used to study changes in the microbial and sensory properties of butter samples during storage. Two‐way ANOVA was employed to statistically analyze the data and examine the significant effects of CSE on the studied characteristics of the butter samples. A comparison of the significant means was carried out using Duncan's multiple range test with a 95% confidence interval (*p* < .05) in SPSS 14. All the tests were performed in triplicate. The results are reported as the mean ± standard deviation (SD).

## 3 RESULTS AND DISCUSSION

### 3.1 Total phenolic content

The TPC of CSE was 11.09 mg GAE/g ethanolic extract, which was higher than those reported by Limam and Mohamed (2019) and Laczkowski et al. (2018), who found that the TPC of chia seed was 4.58 mg GAE/g chia seed flour and 3.59 mg GAE/g ethanolic extract of chia seed, respectively. Moreover, our result was higher than those reported by Tunçil and Çelik (2019), who found that the TPC of white and black chia seeds were 3.52 and 3.42 mg GAE/g defatted chia seeds, respectively. Oliveira‐Alves et al. (2017) reported that TPC content in chia seeds, chia fiber flour, and chia oil was 1.16, 1.11, and 0.02 mg GAE/g, respectively. However, the higher TPC of CSE has been obtained by using a binary mixture of water‐acetone solvent (58.44 mg GAE/g) and a ternary mixture of water–ethanol–acetone solvent (60.96 mg GAE/g) (Alcântara et al., 2019).

The differences in phenolic content of chia seeds reported in various studies and the large extent of lower or higher levels of TPC registered in the mentioned research are due to the differences in varieties and types of samples, the cultivating geographical location of chia seeds, harvesting time, and the extraction methods of phenolic compounds, including type and concentration of the solvent (Scapin et al., 2016). It was reported that the methanolic extract of chia seed shows higher phenolic content compared to other solvent extracts, including water, ethyl acetate, and hexane (Rubavathi et al., 2020). The TPC result of the present research is in line with other studies that indicated CSE is a good source of phenolic compounds, such as chlorogenic acid, caffeic acid, salicylic acid, myricetin, quercetin, and kaempferol, which exhibit antimicrobial and antioxidant activities (Alcântara et al., 2019; Laczkowski et al., 2018; Ullah et al., 2020). It should also be noted that although some other plant extracts, such as garlic, have been reported to contain more TPC than chia extract (Liaqat et al., 2019), their use in butter may not be widely accepted due to their aroma and flavor characteristics.

### 3.2 Analysis of total viable counts

Based on the ANOVA results, all the microbial counts in the butter samples were significantly dependent on CSE percentage and storage time (*p* < .05). As shown in Table 1, the TVC in the control sample (except for the first day of storage) was significantly higher than that of the treatments. The TVC in the butter treatments containing various percentages of the extract was identical on the first day of storage, but significant differences were observed between the treatments after 15 days of storage. In addition, with the increase in the percentage of CSE, TVC reduced significantly in the butter samples (*p* < .05). However, longer storage time significantly increased the TVC of the samples (*p* < .05).

**TABLE 1:** Total viable counts (log CFU/g) in butter samples containing chia seed alcoholic extract during 60 days of refrigerated storage (Mean ± SD).

Treatment	Day				
	1	15	30	45	60
C	3.00 ± 0.00^Da^	3.29 ± 0.00^Ca^	3.69 ± 0.00^Ba^	3.95 ± 0.00^Ba^	4.35 ± 0.00^Aa^
T_1_	3.00 ± 0.00^Ca^	3.26 ± 0.01^Bb^	3.68 ± 0.01^Bb^	3.93 ± 0.01^Bb^	4.31 ± 0.00^Ab^
T_2_	3.00 ± 0.01^Ca^	3.22 ± 0.00^Bc^	3.65 ± 0.00^Bc^	3.73 ± 0.00^Bc^	3.99 ± 0.00^Ac^
T_3_	3.00 ± 0.00^Ca^	3.20 ± 0.00^Bd^	3.56 ± 0.00^Bd^	3.70 ± 0.00^Bd^	3.82 ± 0.00^Ad^
T_4_	3.00 ± 0.00^Ca^	3.13 ± 0.00^Be^	3.55 ± 0.00^Be^	3.69 ± 0.00^Be^	3.74 ± 0.00^Ae^

Abbreviations: C, Control sample, T_1_, Treatment of 0.05% chia extract, T_2_, Treatment of 0.1% chia extract, T_3_, Treatment of 0.25% chia extract, T_4_, Treatment of 0.5% chia extract.

^a–e^Different lowercase letters in each column indicate significant differences between treatments at the level of 5% (*p* < .05).

^A–D^Different uppercase letters in each row indicate significant differences between storage days at the level of 5% (*p* < .05).

On the 60th day of storage, the T_3_ and T_4_ treatments were able to reduce the TVC by 0.53–0.6 log CFU/g compared to the control sample, and the lowest TVC (3.74 log CFU/g) belonged to the T_4_ that contained the highest percentage of the extract. Inhibition of microbial growth could be due to the presence of phytochemicals in chia seeds, including carotenoids, sterols, tocopherols, and phenolic compounds such as quercetin, myricetin, caffeic acid, kaempferol, and chlorogenic acid (Attalla & El‐Husseiny, 2017). Phenolic compounds exhibit antibacterial activity by reacting with cell membranes, inactivating essential enzymes, and degrading or inactivating genetic materials (Kobus‐Cisowska et al., 2019). It has been shown that chia seed has more significant antibacterial activity against Gram‐positive bacteria than Gram‐negative ones (Elshafie et al., 2018).

Thakaeng et al. (2020) reported that the addition of 10% green tea extract to butter reduced the total count by 0.66 log CFU/g compared to the control. It was reported by Asadaii et al. (2020) that the addition of 1.5% turmeric extract, tomato lycopene, and red beet extract powders to butter samples decreased the total counts by 0.16, 0.11, and 0.04 log CFU/g, respectively, compared to the control sample. In other research, the addition of 0.16% chia seed flour to soft cheese reduced the total count by 2.4 log CFU/g compared to the control sample on day 30 of storage (Faid, 2017). Moreover, it was found that by increasing the percentage of chia seed flour in chicken sausage from 2% to 6%, the total count decreased by 0.2 log CFU/g on day 21 of storage (Limam & Mohamed, 2019). Similarly, Attalla and El‐Husseiny (2017) and Zaki (2018), who measured the total bacterial counts of fortified yogurt mousse and camel burger formulated with different levels of chia seeds, respectively, found a significant decrease in microbial counts with increasing chia seed concentration. These results are in agreement with the findings of the present study, which show herbal compounds, especially chia seeds, have antimicrobial characteristics that could inhibit microbial growth during storage.

Regarding the acceptable limit of TVC for butter, which is 5 × 10^4^ CFU/g (Budhkar et al., 2014), none of the studied butter samples in this research exceeded this limit during 60 days of storage.

### 3.3 Analysis of psychrotrophic counts

As can be seen in Table 2, the initial TPC in all samples was in the range of 1.99–2.05 log CFU/g, which is in line with those reported in similar studies (Mehdizadeh et al., 2019). Psychrotrophic counts in both the control sample and the T_1_ treatment containing the lowest percentage of the extract increased significantly from the first to the 60th day of storage by 1.87 log CFU/g. However, in treatments T_2_, T_3_, and T_4_, with higher percentages of the extract, the TPC increased up to the 30th day of storage by 1.25–1.31 log CFU/g and then decreased significantly till the end of the storage period (*p* < .05). These results indicate the inhibitory effects of the CSE on the growth of psychrotrophic microorganisms in butter samples during refrigerated storage.

**TABLE 2:** Total psychrotrophic counts (log CFU/g) in butter samples containing chia seed alcoholic extract during 60 days of refrigerated storage (Mean ± SD).

Treatment	Day				
	1	15	30	45	60
C	2.03 ± 0.00^Ec^	3.05 ± 0.00^De^	3.40 ± 0.00^Ca^	3.60 ± 0.00^Ba^	3.90 ± 0.00^Aa^
T_1_	1.99 ± 0.00^Ee^	3.13 ± 0.01^Dc^	3.33 ± 0.01^Cb^	3.58 ± 0.00^Bb^	3.86 ± 0.00^Ab^
T_2_	2.02 ± 0.01^Ed^	3.16 ± 0.00^Ca^	3.33 ± 0.00^Ab^	3.21 ± 0.00^Bc^	3.00 ± 0.00^Dc^
T_3_	2.04 ± 0.00^Eb^	3.14 ± 0.00^Bb^	3.31 ± 0.00^Ac^	2.96 ± 0.00^Cd^	2.70 ± 0.00^Dd^
T_4_	2.05 ± 0.00^Ea^	3.11 ± 0.00^Bd^	3.30 ± 0.00^Ad^	2.66 ± 0.00^Ce^	2.44 ± 0.00^De^

Abbreviations: C, Control sample, T_1_, Treatment of 0.05% chia extract, T_2_, Treatment of 0.1% chia extract, T_3_, Treatment of 0.25% chia extract, T_4_, Treatment of 0.5% chia extract.

^a–e^Different lowercase letters in each column indicate significant differences between treatments at the level of 5% (*p* < .05).

^A–E^Different uppercase letters in each row indicate significant differences between storage days at the level of 5% (*p* < .05).

Moreover, T_3_ and T_4_ were able to reduce the TPC by 1–1.5 log CFU/g on days 45 and 60 of storage compared to the control sample. This was due to the presence of phenolic compounds in the extract, such as flavonoids, including kaempferol and quercetin, and caffeic acid whose antimicrobial effects have been proven (Kobus‐Cisowska et al., 2019). In addition, the considerable amount of caryophyllene in CSE has strong antibacterial effects against psychrotrophic bacterial species such as *Pseudomonas aeruginosa*, *Bacillus cereus*, and *Bacillus subtilis* (Elshafie et al., 2018).

Mehdizadeh et al. (2019) studied the effects of hydroalcoholic extract of walnut kernel septum membranes on butter shelf life and reported that the treatment containing 0.5% extract reduced psychrotrophs by 2 log CFU/g compared to the control on day 90 of storage. In another study by Zaki (2018), the addition of 5% chia seed to a camel burger decreased the psychrotrophic bacterial count by 0.98 log CFU/g compared to the control sample on day 12 of storage. These results are in agreement with the findings of the present research regarding the similar effects of herbal compounds on reducing the number of psychrotrophs.

The maximum acceptable limit of TPC in butter is 10^4^ CFU/g, and in this research, none of the studied samples exceeded this limit during the 60‐day refrigerated storage.

### 3.4 Analysis of coliform counts

On the first day of storage, the coliform counts in all butter samples were the same (Table 3). The number of coliforms increased in the control sample by 2.8 log CFU/g from the 15th to the 60th day of storage. However, it significantly decreased in the treatments that contained the chia extract (*p* < .05). Furthermore, longer storage time led to significant increases in the number of coliforms in the control sample, but the number of coliforms was reduced in the treatments containing the extract up to the 60th day of storage (*p* < .05).

**TABLE 3:** Coliform counts (log MPN/g) in butter samples containing chia seed alcoholic extract during 60 days of refrigerated storage (Mean ± SD).

Treatment	Day				
	1	15	30	45	60
C	1.17 ± 0.00^Ea^	2.04 ± 0.01^Da^	2.92 ± 0.02^Ca^	3.35 ± 0.02^Ba^	3.96 ± 0.01^Aa^
T_1_	1.17 ± 0.00^Aa^	1.17 ± 0.00^Ab^	0.96 ± 0.00^Bb^	0.96 ± 0.00^Bb^	0.63 ± 0.00^Cb^
T_2_	1.17 ± 0.00^Aa^	0.96 ± 0.00^Bc^	0.96 ± 0.00^Bb^	0.36 ± 0.00^Cc^	0.00 ± 0.00^Dc^
T_3_	1.17 ± 0.00^Aa^	0.63 ± 0.00^Bd^	0.44 ± 0.00^Cc^	0.00 ± 0.00^Dd^	0.00 ± 0.00^Dd^
T_4_	1.17 ± 0.00^Aa^	0.46 ± 0.00^Be^	0.00 ± 0.00^Cd^	0.00 ± 0.00^Cd^	0.00 ± 0.00^Cc^

Abbreviations: C, Control sample, T_1_, Treatment of 0.05% chia extract, T_2_, Treatment of 0.1% chia extract, T_3_, Treatment of 0.25% chia extract, T_4_, Treatment of 0.5% chia extract.

^a–e^Different lowercase letters in each column indicate significant differences between treatments at the level of 5% (*p* < .05).

^A–E^Different uppercase letters in each row indicate significant differences between storage days at the level of 5% (*p* < .05).

Coliform enumeration decreased in T_1_ by 0.5 log CFU/g during 60‐day storage, and no coliforms were detected in T_2_, T_3_, and T_4_ after 60, 45, and 30 days of refrigerated storage, respectively. In other words, treatments T_2_, T_3_, and T_4_ were able to decrease the coliform counts by 3–4 log CFU/g on days 45 and 60 of storage compared to the control sample. The antimicrobial activity of chia seeds can be explained by the considerable presence of flavonoids and caryophyllene that have antimicrobial properties against some pathogenic bacteria, such as *E. coli* and *Klebsiella pneumoniae*, which can be among coliforms as well (Elshafie et al., 2018). Furthermore, the inhibition of bacterial growth can be attributed to the extract concentration and the chemicals present in it that led to a rupture of the bacterial cell membrane (Mehdizadeh et al., 2019).

In research by Asadaii et al. (2020), the addition of 1.5% turmeric extract, red beet extract, and lycopene powders to butter decreased coliform counts by 0.33, 0.02, and 0.02 log CFU/g compared to the control sample, respectively. However, in the present study, CSE had greater effects on the reduction of coliforms at much lower concentrations. Similarly, coliforms were not detected in yogurt mousse fortified with chia seeds during 8 days of storage at 6°C (Attalla & El‐Husseiny, 2017). Consistent with our results, Mikdame et al. (2020) reported that the addition of olive mill wastewater and olive pomace at a concentration of 8 mg/100 g to butter under an ambient temperature led to a 0.84 log CFU/g reduction in coliform counts on day 90 of storage.

Considering the maximum acceptable limit of coliforms in butter (10 CFU/g), it was observed that the coliform counts in the control sample on all storage days and in the treatments containing the chia extract on the first day of storage exceeded the acceptable limit. This could be due to contamination of butter by washing water, equipment, and other sources during manufacturing and packaging. However, adding CSE caused coliform counts to decrease below the acceptable limit in T_1_ after 30 days and in treatments T_2_, T_3_, and T_4_ after 15 days of storage.

### 3.5 Analysis of mold and yeast counts

As shown in Table 4, the initial number of molds and yeasts ranged from 0.95 to 1.15 log CFU/g in butter samples, which is in accordance with similar studies (Mahmoudi et al., 2019; Naurzbayeva et al., 2023). The mold and yeast counts in the control sample were significantly higher than the other treatments, except for the first day of storage. Moreover, the use of higher concentrations of CSE significantly decreased the number of molds and yeasts (*p* < .05). These counts increased gradually in the control sample along with the storage time, but these enumerations showed a descending trend in T_2_, T_3_, and T_4_ treatments after 15 days of storage (*p* < .05). Moreover, T_2_, T_3_, and T_4_ were able to reduce the number of molds and yeasts on storage days 30, 45, and 60 by 1.5–2.6 log CFU/g compared to the control sample.

**TABLE 4:** Mold and yeast counts (log CFU/g) in butter samples containing chia seed alcoholic extract during 60 days of refrigerated storage (Mean ± SD).

Treatment	Day				
	1	15	30	45	60
C	0.95 ± 0.00^Ec^	2.11 ± 0.02^Da^	3.20 ± 0.02^Ca^	3.43 ± 0.04^Ba^	3.60 ± 0.02^Aa^
T_1_	1.05 ± 0.05^Eb^	2.11 ± 0.02^Ba^	2.19 ± 0.01^Ab^	2.05 ± 0.02^Cb^	1.83 ± 0.06^Db^
T_2_	1.15 ± 0.07^Ea^	1.95 ± 0.00^Ab^	1.65 ± 0.00^Bc^	1.35 ± 0.08^Cc^	1.25 ± 0.00^Dc^
T_3_	1.05 ± 0.05^Db^	1.65 ± 0.08^Ac^	1.37 ± 0.00^Bd^	1.07 ± 0.00^Cd^	0.95 ± 0.00^Ed^
T_4_	0.95 ± 0.00^Bc^	1.47 ± 0.07^Ad^	0.95 ± 0.00^Be^	0.95 ± 0.00^Be^	0.95 ± 0.00^Bd^

Abbreviations: C, Control sample, T_1_, Treatment of 0.05% chia extract, T_2_, Treatment of 0.1% chia extract, T_3_, Treatment of 0.25% chia extract, T_4_, Treatment of 0.5% chia extract.

^a–e^Different lowercase letters in each column indicate significant differences between treatments at the level of 5% (*p* < .05).

^A–E^Different uppercase letters in each row indicate significant differences between storage days at the level of 5% (*p* < .05).

The presence of high humidity during storage and the permeability of packaging material may support the growth of molds on the surface of butter. Also, the growth of yeasts, such as Saccharomyces, Candida, and Torulopsis, can cause acid production and spoilage of butter (Budhkar et al., 2014). It has been shown that chia seed essential oil has antifungal effects, especially against *Aspergillus fumigatus*, *Penicillium expansum*, *Monilinia laxa*, and *Monilinia fructigena* (Elshafie et al., 2018). Human pathogens, including yeasts such as *Candida albicans*, are also sensitive to the inhibitory effects of phenolic acids, flavonoids, tannins, and anthocyanins present in CSE (Kobus‐Cisowska et al., 2019).

In accordance with the results of the present research, Mikdame et al. (2020) reported that the addition of 8 mg/100 g of olive oil by‐products to butter decreased the mold and yeast counts by 1.15 and 1.23 log CFU/g on day 90 of storage at 25°C and 60°C, respectively. Thakaeng et al. (2020) also added 10% (w/w) green tea extract to butter and reported that the mold and yeast counts decreased by 0.52 log CFU/g compared to the control sample. Also, it has been reported that the addition of carrot pomace‐based emulsion to pasteurized butter resulted in a reduction of mold and yeast counts (up to 1.47–2.47 log CFU/g) compared to the control during 60 days of storage at 4°C (Naurzbayeva et al., 2023). Furthermore, Faid (2017) reported that molds and yeasts were not detected in soft cheese fortified with 8%–16% chia seed flour during 30 days of storage at 5°C, and chia addition to these treatments led to a 4.8 log CFU/g decrease in mold and yeast counts in comparison with the control sample. Limam and Mohamed (2019) also studied the effect of chia seed flour on chicken sausage stored at 4°C for 21 days and reported that increasing the percentage of chia seed flour from 2% to 6% reduced mold and yeast counts by 0.2 log CFU/g on day 21 of storage. The results of these studies on the effectiveness of the studied compounds in reducing mold and yeast counts are in agreement with those of this research and suggest that CSE has antimicrobial effects against molds and yeasts and can decrease their growth rate.

The maximum acceptable limit of mold and yeast count in butter is 10^2^ CFU/g. In this research, the mold and yeast counts in the control sample and T1 exceeded this limit after the first day of storage, but in other treatments (T_2_, T_3_, and T_4_) it was below the acceptable limit during the 60 days of storage.

### 3.6 Analysis of *E. coli* and *S. aureus* detection

The results of the detection and enumeration of *E. coli* and *S. aureus* in the control samples and the treatments containing the CSE during 60‐day refrigerated storage were negative, and *E. coli* and *S. aureus* were not detected in the samples, which is in compliance with the current standards. The absence of these pathogenic bacteria in the product is required, and it indicates the satisfactory quality of butter (Budhkar et al., 2014). This is due to the application of pasteurization, good hygienic practices, and maybe the presence of chia seed phenolic compounds in butter. Although these pathogenic bacteria were not isolated in the present study, the antibacterial activity of chia seed methanolic extract has been reported against *S. aureus* with an inhibition zone of 25 mm (Rubavathi et al., 2020). Dark chia seeds were used in the present study, and it has been reported that the synthesized silver nanoparticles of dark chia seeds had more antimicrobial activity against both *E. coli* (with 18.5 mm inhibition zone) and *S. aureus* (with 15.4 mm inhibition zone) in comparison with white chia seeds (Hernández‐Morales et al., 2019).

Mikdame et al. (2020) reported a total absence of *S. aureus* in all studied butter samples containing olive oil by‐products during storage. However, contrary to the findings of the present research, Mehdizadeh et al. (2019) and Naurzbayeva et al. (2023) reported the initial *S. aureus* count of 1.19 and 1.16 log CFU/g in traditional and pasteurized butter samples, respectively, which has been increased during 90 and 60 days of storage to above 8.33 and 6.6 log CFU/g in both the control and treatments. Also, *E. coli* was detected in traditional butter containing *Ziziphora cliniopodioides* essential oil in the range of 0.09 to 3.68 log CFU/mL during 10 days of refrigerated storage (Mahmoudi et al., 2019). Thus, it seems necessary to use good manufacturing practices along with the use of natural antimicrobial compounds against these pathogenic bacteria in butter.

### 3.7 Analysis of sensory properties

**FIGURE 1:**
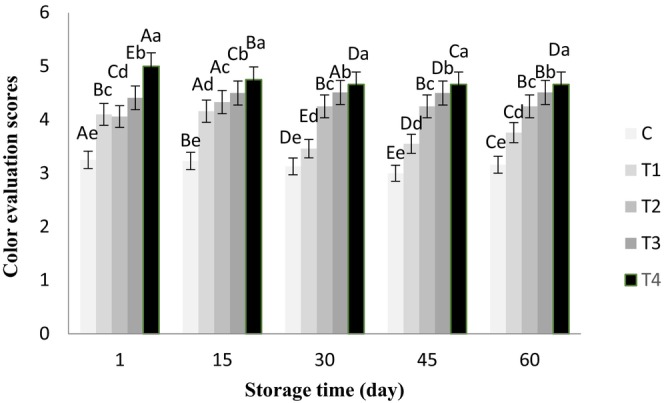
Color scores of butter samples containing chia seed extract during 60 days of refrigerated storage. ^a–e^Different lowercase letters in each day indicate significant differences between treatments at the level of 5% (*p* < .05). ^A–E^Different uppercase letters in each treatment indicate significant differences between storage days at the level of 5% (*p* < .05). C, Control sample, T_1_, Treatment of 0.05% chia extract, T_2_: Treatment of 0.1% chia extract, T_3_, Treatment of 0.25% chia extract, T_4_, Treatment of 0.5% chia extract.

**FIGURE 2:**
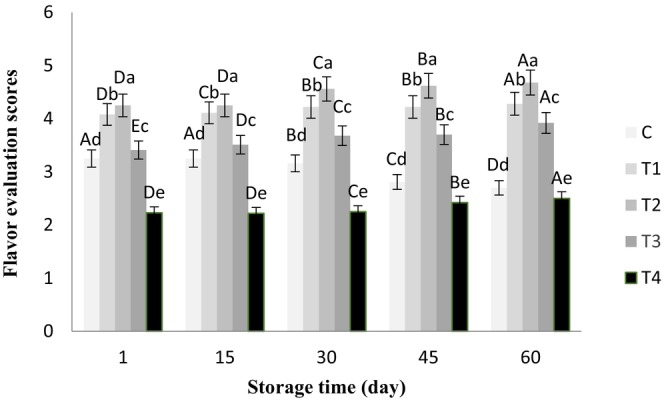
Flavor scores of butter samples containing chia seed extract during 60 days of refrigerated storage. ^a–e^Different lowercase letters in each day indicate significant differences between treatments at the level of 5% (*p* < .05). ^A–E^Different uppercase letters in each treatment indicate significant differences between storage days at the level of 5% (*p* < .05). C, Control sample, T_1_, Treatment of 0.05% chia extract, T_2_, Treatment of 0.1% chia extract, T_3_, Treatment of 0.25% chia extract, T_4_, Treatment of 0.5% chia extract.

**FIGURE 3:**
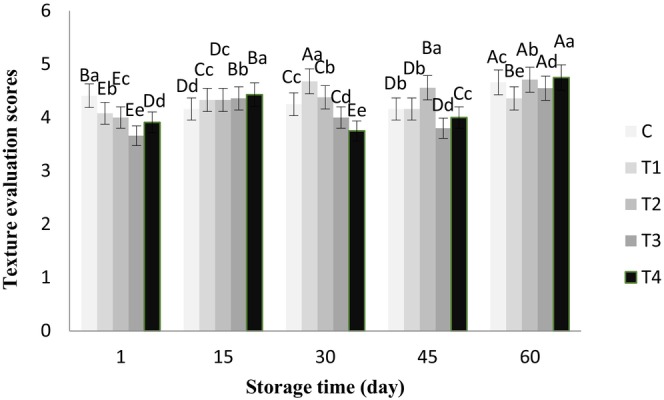
Texture scores of butter samples containing chia seed extract during 60 days of refrigerated storage. ^a–e^Different lowercase letters in each day indicate significant differences between treatments at the level of 5% (*p* < .05). ^A–E^Different uppercase letters in each treatment indicate significant differences between storage days at the level of 5% (*p* < .05). C, Control sample, T_1_, Treatment of 0.05% chia extract, T_2_, Treatment of 0.1% chia extract, T_3_, Treatment of 0.25% chia extract, T_4_, Treatment of 0.5% chia extract.

**FIGURE 4:**
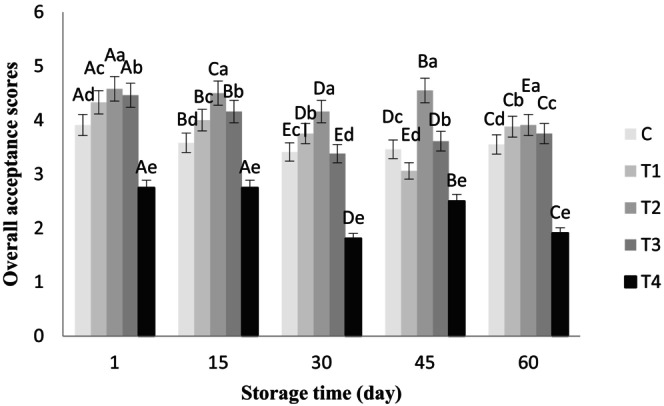
Overall acceptance scores of butter samples containing chia seed extract during 60 days of refrigerated storage. ^a–e^Different lowercase letters in each day indicate significant differences between treatments at the level of 5% (*p* < .05). ^A–E^Different uppercase letters in each treatment indicate significant differences between storage days at the level of 5% (*p* < .05). C, Control sample, T_1_, Treatment of 0.05% chia extract, T_2_, Treatment of 0.1% chia extract, T_3_, Treatment of 0.25% chia extract, T_4_, Treatment of 0.5% chia extract.

The ANOVA results showed that chia seed alcoholic extract had no significant effect on the aroma of the butter samples, and all of them gained a good score of >4 during storage. However, the effects of the treatments and storage time were significant on color, flavor, texture, and overall acceptance of all the butter samples (*p* < .05), which are shown in Figures 1, 2, 3, 4, respectively.

Higher percentages of the chia extract significantly increased the color score of the butter treatments (*p* < .05). Longer storage time decreased the color score of the control sample and T_1_ (with 0.05% extract) (*p* < .05). During the storage period, the highest color score belonged to T_4_ (butter +0.5% extract), and the lowest color score (in the range of 2.58–3.25) was obtained in the control sample (*p* < .05). In general, the lower color scores were assigned to the control sample and T1, which had higher microbial counts compared to the other treatments. It is known that molds, yeasts, and bacteria, including coliforms, are responsible for undesirable butter color changes (Ahmed et al., 2016).

Higher percentages of chia extract in the butter samples also improved the flavor scores (except for T_4_) compared to the control sample (*p* < .05). Over storage time, the flavor score decreased in the control sample but increased in the treatments containing the chia extract. The flavor of the control sample was not acceptable after 30 days, which could be due to the microbial growth in butter. It has been reported that Pseudomonas spp. grow well at refrigerator temperatures and produce putrid or lipolytic flavors in 10 days (Budhkar et al., 2014). However, the treatment that included 0.1% extract (T_2_) had the highest flavor score and acceptable quality during storage, and the treatment containing 0.5% extract (T_4_) obtained the lowest flavor score. Apart from the ingredients used in the butter formulation and the storage time and temperature, which can influence butter flavor, encapsulating chia seed extract can reduce its effects on the sensory characteristics of some dairy products (Hosseini et al., 2023).

Texture scores increased significantly over time compared to the first day of storage (*p* < .05). On the first day of storage, the highest (4.16) and the lowest (3.91) scores for texture belonged to the control sample and T_4_, respectively, but on the 60th day of storage, the highest (4.75) sensory score for texture belonged to T_4_ and the lowest (4.36) was assigned to T_1_ (with 0.05% extract), which was significantly different from other treatments (*p* < .05). The sensory scores for texture given to butter samples did not follow a specific and constant trend in this research. This could be due to the storage temperature and oily compounds present in chia seed extract that greatly influence the hardness of butter.

The overall acceptance scores decreased in all the samples along with storage time. Moreover, the highest percentage of chia extract in T_4_ significantly decreased the overall acceptance score, but its lower percentages increased the scores of the other treatments compared to the control sample (*p* < .05). During the storage period, the highest score for overall acceptance belonged to T_2_ (with 0.1% extract) and the lowest (in the range of 1.81–2.75) to T_4_ (with 0.5% extract), which were significantly different from other treatments (*p* < .05). These results are close to those obtained by Zaki (2018), who found that camel burgers formulated with 3% chia seeds showed a higher score in sensory attributes, including texture, flavor, and overall acceptability, compared to their lower (1%) and higher (5%) concentrations.

Mahmoudi et al. (2019) revealed that butter containing *Ziziphora cliniopodioides* essential oil had desirable sensory scores at concentrations of 300–900 ppm, but the lowest sensory acceptance was observed at the highest concentration. Similarly, Kibui et al. (2018) added chia seeds to yogurt and reported that the panelists preferred the texture, flavor, color, and appearance of the control and the yogurt containing 1.5% chia seeds compared to the samples with 2.5% and 3.5% chia seeds. In addition, Feizi et al. (2021), who used chia seed gum (CSG) as a stabilizer in ice cream, found that the treatment containing 0.2% CSG was the best one in terms of sensory properties, and it had a smooth texture without off‐flavor. These results are in line with the findings of the present research. However, Attalla and El‐Husseiny (2017) reported that yogurt mousse fortified with 3% chia seeds obtained the highest scores for all sensory attributes during storage, and yogurt mousse with 1% chia showed the lowest scores. Furthermore, Hosseini et al. (2023), who compared the effect of free chia seed extract (F‐CSE) with encapsulated CSE (at 1.5% and 3.0% w/w) on the sensory properties of ricotta cheese, declared that the addition of F‐CSE to the cheese formulation led to a decrease in all sensory scores, but encapsulated CSE treatments did not have a negative effect on sensory acceptance. Ullah et al. (2020) evaluated the effect of chia oil microcapsules in butter and reported non‐significant findings in color, flavor, and texture of all the experimental samples, and their sensory characteristics were similar to the control. These differences could be related to the variation in the studied dairy products, different application forms, non‐encapsulated or encapsulated, concentrations, and types of chia seeds used.

## 4 CONCLUSION

The results of this research indicated that the use of chia seed alcoholic extract in butter, especially at higher concentrations, significantly reduced TVC, TPC, coliforms, mold, and yeast counts in treatments compared to the control sample. In addition, longer storage time significantly increased all microbial counts in the control sample, but this increase had a slower trend in the treatments containing chia seed extract and even coliforms, and molds and yeasts decreased over time in T_3_ and T_4_, which had higher concentrations of chia seed extract. Moreover, *E. coli* and *S. aureus* were not detected in all the butter samples. Evaluation of sensory properties revealed that the treatments containing 0.1% and 0.25% of the chia seed extract (T_2_ and T_3_) had acceptable and satisfactory quality and were selected as the superior ones with respect to all sensory properties during the 60‐day storage. So, regarding the considerable content of phenolic compounds and antimicrobial activity of chia seed alcoholic extract, it can be used to improve the microbial quality and sensory properties of pasteurized sweet cream butter and probably other dairy products.

## AUTHOR CONTRIBUTIONS

**Zahra Sadri‐Saeen:** Conceptualization (equal); data curation (equal); formal analysis (equal); investigation (equal); methodology (equal); resources (lead); software (lead); visualization (equal); writing – original draft (equal). **Mohammadreza Khani:** Conceptualization (equal); data curation (equal); formal analysis (equal); investigation (equal); methodology (equal); project administration (lead); supervision (lead); validation (equal); visualization (equal); writing – original draft (equal); writing – review and editing (equal). **Vajiheh Fadaei:** Conceptualization (equal); methodology (equal); validation (equal); writing – review and editing (equal).

## FUNDING INFORMATION

No funding was received for conducting this study.

## CONFLICT OF INTEREST STATEMENT

The authors declare that they have no conflict of interest.

## ETHICS STATEMENT

This study does not involve any human or animal testing.

## Data Availability

Data will be made available on request.
